# Distribution of chronic wasting disease (CWD) prions in tissues from experimentally exposed coyotes (*Canis latrans*)

**DOI:** 10.1371/journal.pone.0327485

**Published:** 2025-07-09

**Authors:** Nancy Ho, Reece McGinn, Paulina Soto, Terry R. Spraker, Justin Fischer, Kurt VerCauteren, Tracy Nichols, Rodrigo Morales

**Affiliations:** 1 Department of Neurology, The University of Texas Health Science Center at Houston, Houston, Texas, United States of America; 2 Centro Integrativo de Biologia y Quimica Aplicada (CIBQA). Universidad Bernardo O’Higgins. Santiago, Chile; 3 Colorado State University Diagnostic Medical Center, College of Veterinary Medicine and Biomedical Sciences, Colorado State University. Fort Collins, Colorado, United States of America; 4 United States Department of Agriculture, Animal Plant Health Inspection Service, Wildlife Services. Fort Collins, Colorado, United States of America; 5 United States Department of Agriculture, Animal Plant Health Inspection Service, Veterinary Services. Fort Collins, Colorado, United States of America; Colorado State University College of Veterinary Medicine and Biomedical Sciences, UNITED STATES OF AMERICA

## Abstract

Cervids susceptible to chronic wasting disease (CWD) are sympatric with multiple other animal species that can interact with infectious prions. Several reports have described the susceptibility of other species to CWD prions, or their potential to transport them. One of these species is the coyote (*Canis latrans*), which has been previously shown to pass transmission-relevant prion titers in their feces for at least three days after ingesting prion-positive brain material. The current study followed up on these findings and evaluated the distribution of prions in multiple tissues from the same coyotes. Our results show that prions persist in the digestive tract of prion-exposed coyotes five days after exposure. Moreover, prion seeding activity was identified in other tissues, including lymph nodes and lungs. These results provide additional information about the dynamics of CWD prions in the environment and show the initial fate of prions after ingestion by a canid species that is a carnivorous predator and scavenger.

## Introduction

Chronic wasting disease (CWD) is a transmissible spongiform encephalopathy (TSE) affecting members of the deer family [1,2]. Each year, an increasing number of states in the United States (U.S.) and provinces in Canada report cases of CWD in both, farmed and wild cervid populations (https://www.usgs.gov/media/images/distribution-chronic-wasting-disease-north-america-0). At present, 36 U.S. states and 5 Canadian provinces have reported cases of CWD, as have countries in Asia (South Korea) and Europe (Norway, Finland, and Sweden). TSEs, including CWD, are invariably fatal [1,2]. Currently, there are no effective treatments for CWD and controlling its spread has been challenging.

The mechanisms explaining the rapid spread of CWD are not fully understood and many factors likely contribute to its epidemiology. Anecdotal and experimental evidence suggest that animals can get infected through vertical or horizontal mechanisms, with the latter being the most accepted [3,4]. Along this line, the exposure of susceptible animals to prion-contaminated fomites or contaminated soil appears to play a relevant role in the transmission of prion pathogens [4–6]. Human activities, such as the transport of live animals and the movement of carcasses to areas otherwise free of CWD, have also been proposed as routes for the spread of this disease. Unfortunately, CWD can be easily transmitted between different cervid species, enhancing the possibilities of prion strain variation [3,7–9].

Animals at the terminal stage of CWD die or become easy prey for predators. Carcasses of diseased cervids can become available to scavengers. Although these interactions are potentially relevant for CWD spread, no cases of transmission in non-cervid species have been described in natural scenarios. Nevertheless, the potential interspecies transmission of CWD to non-cervid species has been extensively explored in natural hosts and transgenic mice expressing the prion protein sequences of sympatric species [10–15]. Although positive transmissions have been described in many cases, they mostly result after intracerebral administrations of high quantities of prions and display long incubation periods suggestive of strong species barriers. The latter proposes that the inter-species transmission of prions to non-cervid species is difficult but not impossible.

Despite the strong species barriers, predators and scavengers may still be relevant as spreaders of infectious prion particles. In fact, both experimental research and natural observations indicate that insects, parasites, and various wild and domestic animals that co-exist with cervids can act as passive carriers of CWD prions [16–23]. For example, coyotes (*Canis latrans*) are relevant deer predators and scavengers. Previous studies showed that coyotes fed with an elk brain infected with CWD passed prions through their feces for at least three days after ingestion [23]. Similar observations were reported for cougars (*Puma concolor*) [21]. In contrast, crows (*Corvus brachyrhynchos*) passed prions for only few hours after ingesting infected materials [22]. Importantly, previous research in coyotes and cougars suggest that excreted prions contain decreased infectivity titers compared with the ingested material, suggesting that infectious particles are being retained in tissues within these animals, or degraded. In natural settings, prions have been identified in scat from multiple species of sympatric animals [20]. Overall, data indicates that predators and scavengers can excrete infectious prions after ingesting contaminated tissues. This, in turn, may contribute to the environmental spread of prions.

As mentioned above, the decreased titers of infective prions released in excreta may be partially explained by degradation of infectious particles by the animal that ingested infected tissues. A presumably small proportion of prions may also be incorporated in tissues from these animals. Previous reports in white-tailed deer (*Odocoileus virginianus*) and Syrian hamsters (*Mesocricetus auratus*) suggest that some of these prions may get sequestered in lymphoid tissues shortly after ingestion [24,25]. Similar prion retention phenomena may occur in predators and scavengers.

This study followed up a previous report by Nichols *et al*. [23] by analyzing the tissues from coyotes shortly (5 days) after they were exposed to CWD prions. These evaluations provide additional data to inform the understanding of the dynamics of CWD prions in natural scenarios, and further unveil the fate of prions in non-cervid animal species.

## Materials and methods

### Tissues samples

*Postmortem* tissues samples from the previous study [23] (5 coyotes; 2 males and 3 females), were used in this study. Inoculation methods for these animals were previously described [23]. Briefly, coyotes received a single CWD-positive (*n* = 3) or CWD-free (*n* = 2) brain meal and feces were daily collected from 1 day before treatment to 5 days after treatment. *Postmortem* samples from these animals were collected five days post-exposure, stored at −80 °C, and transported from USDA facilities to The University of Texas Health Science Center at Houston (UTHealth-Houston) for further analyses. Pieces of coyote tissues (e.g., intestine, cecum, liver, tonsil, mesenteric lymph nodes, spleen, brain, lung and heart) were cut and homogenized at 20% weight/volume (w/v) in phosphate buffer saline (PBS, Hyclone PBS, GE Healthcare Life Science) supplemented with a protease inhibitors cocktail (cOmplete^TM^, EDTA-free Protease Inhibitor Cocktail, Roche). Gastrointestinal tissues (cecum, intestine) were washed with PBS before homogenization to remove fecal matter. Fecal samples were treated as mentioned above. The homogenization process was conducted using a Precellys ® 24 homogenizer following established protocols for soft or hard tissues, as previously reported [16]. The resulting homogenates were placed on ice in between homogenization cycles and stored at −20 °C until used. Animal #136 from the original study [23] was not sampled, so the tissues from this coyote were not available for PMCA analysis. Fecal samples were not subjected to any pretreatment before PMCA analyses as described in our previous publication [26].

### PMCA substrate

Substrate for PMCA was prepared using brains of tg1536^+/+^ (homozygous) mice [27]. All animal procedures were covered by protocol number AWC-22–0039, approved by the Animal Welfare Committee at UTHealth-Houston. Mice were humanly euthanized by CO_2_ inhalation and brains collected after cardiac perfusion with cold PBS (Hyclone PBS, GE Healthcare Life Sciences) supplemented with 5mM EDTA (Promega). Perfused brains were stored at −80 °C until use. The brains were homogenized at a concentration of 10% w/v in PMCA conversion buffer (PBS, supplemented with 1% Triton X-100 (Sigma-Aldrich), 150 mM NaCl (Sigma-Aldrich) and a protease inhibitor cocktail). Homogenates were then centrifuged at 800 x *g* and 4 °C for 40 seconds. The supernatants were collected, vortexed, aliquoted, and stored at −80 °C. Right before use, substrate aliquots were thawed in ice and supplemented with 5 mM EDTA (Promega) and digitonin 0,025% (Invitrogen).

### PMCA procedure

Ten µL of the 20% w/v coyote tissues and fecal samples homogenates were mixed with 90 µL of PMCA substrate. The PMCA procedure was performed as extensively described in our previous publication [28] and adapted for the detection of CWD prions [16,29,30]. In summary, a first PMCA round was conducted by subjecting the sample/substrate mixture to 144 cycles of incubation and sonication (each PMCA cycle included 29 minute and 40 seconds of incubation, and 20 seconds of sonication). The resulting materials after a first round of PMCA were submitted to two additional rounds of PMCA (96 cycles each) by mixing 10 µL of the PMCA products of each round with new 90 µL of PMCA substrate. As positive controls, each PMCA reaction set (testing approximately 10 samples) included serial dilutions of CWD-infected brains of known PMCA activity. Four unseeded reactions (NC) were used as negative controls. PMCA assays were conducted in a blind manner by two researchers, in triplicate. Results were expressed as positive or negative for each replicate.

### Proteinase K (PK) treatment

To evaluate the potential presence of disease-associated prion protein (PrP^Sc^) in a given sample, 20 µL of PMCA products were treated with 100 µg/mL PK (Sigma-Aldrich) at 37 °C and 450 rpm shaking for 80 minutes using an Eppendorf thermomixer. PK reactions were stopped by adding 25 µL of 2X Laemmli LDS sample buffer (BioRad) or NuPAGE™ LDS Sample Buffer (4X) and exposure to 90°C for 10 minutes. The resulting materials were examined by western blotting.

### Electrophoresis and western blotting

Electrophoresis was performed by two different approaches. First, we used 12% Bis-Tris gels (Invitrogen) and MOPS Buffer (NuPAGE^TM^) run at 80 V for 20 minutes, then 140 V for 1 hour 10 minutes. The second approach used 12% Mini-PROTEAN^®^ TGX^TM^ Precast Gels (Bio-Rad) and 10X Tris/Glycine/SDS buffer at 300 V for 15–25 minutes. We have not observed major differences between these two methods, and they were used depending on the preference of the manipulators. Next, fractioned proteins were transferred onto a 0.45 µm nitrocellulose membrane (Amersham Protran) immersed in transfer buffer using the Trans-Blot Turbo Transfer System (Bio-Rad) following preprogrammed protocols (2.5 A, up to 25 V) for 7 minutes. Membranes were blocked using 10% (w/v) non-fat milk (Lab Scientific bioKEMIX) for 15 minutes, then membranes were incubated with the primary monoclonal 8H4 antibody (Sigma-Aldrich) diluted at 1:5,000 overnight at 4 °C. The same membranes were probed with secondary polyclonal Anti-Mouse IgG (whole molecule) – Peroxidase antibody produce in sheep (Sigma-Aldrich) diluted at 1:3,000 for 1 hour at room temperature. Membranes were washed three times for 10 minutes with PBST – 0.05% after being incubated with each antibody. Lastly, the membranes were developed using ECL (Cytiva Amersham) in a BioRad dark chamber to visualize the results.

## Results

**Fig 1:**
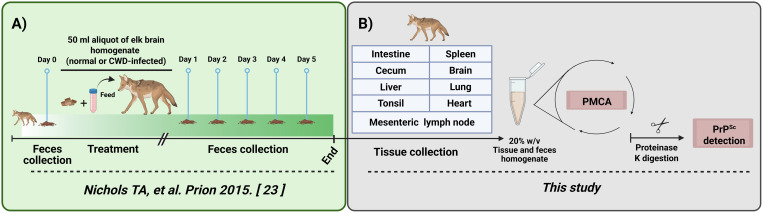
Experimental design describing the administration of CWD-contaminated tissues to coyotes, and sample collection. A) Schematics of the experimental design described in Nichols *et al*. [23]. Three coyotes (*Canis latrans*) were fed a brain extract containing CWD prions from elk, and feces were collected one day before and five days after treatments. Two additional coyotes were treated with a CWD-free elk brain and feces collected as explained before. B) Following 5 days of feces collection, coyotes were sacrificed and multiple tissues were collected and tested for prion content by PMCA.

We built upon a previous study published by Nichols *et al*. [23] describing the excretion of CWD prions by coyotes after ingesting prion contaminated materials. Specifically, the original study included feeding coyotes with elk brain meals prepared from CWD-positive and CWD-free elk. Fecal materials from these coyotes were collected one day before, and daily for five days after elk brain exposure, and the presence of prions in these samples were measured by means of the PMCA technique, and bioassays (Fig 1A). In summary, the results obtained in the previous study showed that coyotes shed prions in their excreta for at least three days after exposure. The prions present in these samples were able to sustain prion infectivity after inoculation into transgenic mice expressing the elk prion protein [23]. Importantly, the incubation periods obtained in the previous study suggested that the prion titers decrease after passage through the coyotes’ gastrointestinal tracts. This reduction could be due to the degradation of infectious particles. Although presumably not playing a major role in these decreased infectivity titers, absorption and distribution of CWD prions within the coyotes’ tissues was also suspected. To analyze the latter, *postmortem* tissues were sampled from these experimental canids and tested through the PMCA technique (Fig 1B).

It is important to note that here we used a different PMCA setting compared to that used in the original study. Specifically, brains from tg1536 mice [27] were utilized as substrate for the assay, and results were interpreted at the third PMCA round. We observed that the amplification of some CWD prions, regardless of their origin, is similar using Tg1536 (deer) and Tg5037 (elk) substrates (data not shown). This is the case for elk prions and replication in deer substrate. Considering the abundance of Tg1536 substrate at the moment of performing the experiments, we opted to use this specific source. This was also considered as our project aimed for detection, and not to study species or prion strain specific mechanisms. This methodology allowed us to address the specific question of evaluating the presence of CWD prions in tissues and excreta of exposed coyotes.

**Fig 2:**
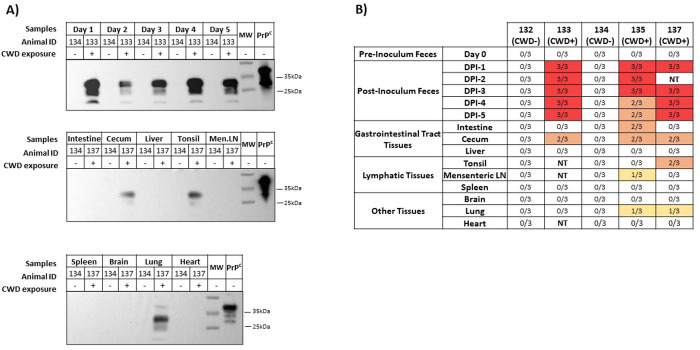
PMCA analyses of fecal samples and tissues from coyotes exposed to CWD prions. A) Western blots depicting PMCA products (third round) of representative fecal samples and coyote tissues exposed (+), or not (-), to CWD prions. All samples were treated with proteinase K, as explained in Material and Methods, with the exception of “PrP^C^” (brain extracts from tg1536 mice) that were used to control molecular weights and antibody reactivity. Numbers at the right represent molecular weights. The samples depicted in this panel are representative of coyotes exposed (#133, upper panel; #137, middle and lower panel) or not (#134) to CWD prions. B) Summary of the results collected for this study. Each sample was tested in triplicate, by two researchers. “CWD +” and “CWD –“represents animals exposed (+), or not (-), to CWD prions. Positive results in PMCA are color-filled in the table (red: 3/3; orange: 2/3; yellow: 1/3; no fill: 0/3). Fecal samples are denoted by “Day”, and numbers indicate the collection day after treatment (Day 0 represent control samples collected before treatments). N/T: not tested. Coyotes #133, #135 and #137 were fed a brain homogenate containing CWD prions. Coyotes #132 and #134 were fed a CWD-free elk brain homogenate. Raw data for this figure are available in S1 Raw Image. Additional replicates for the samples tested here are shown in S1 Fig.

**Fig 3:**
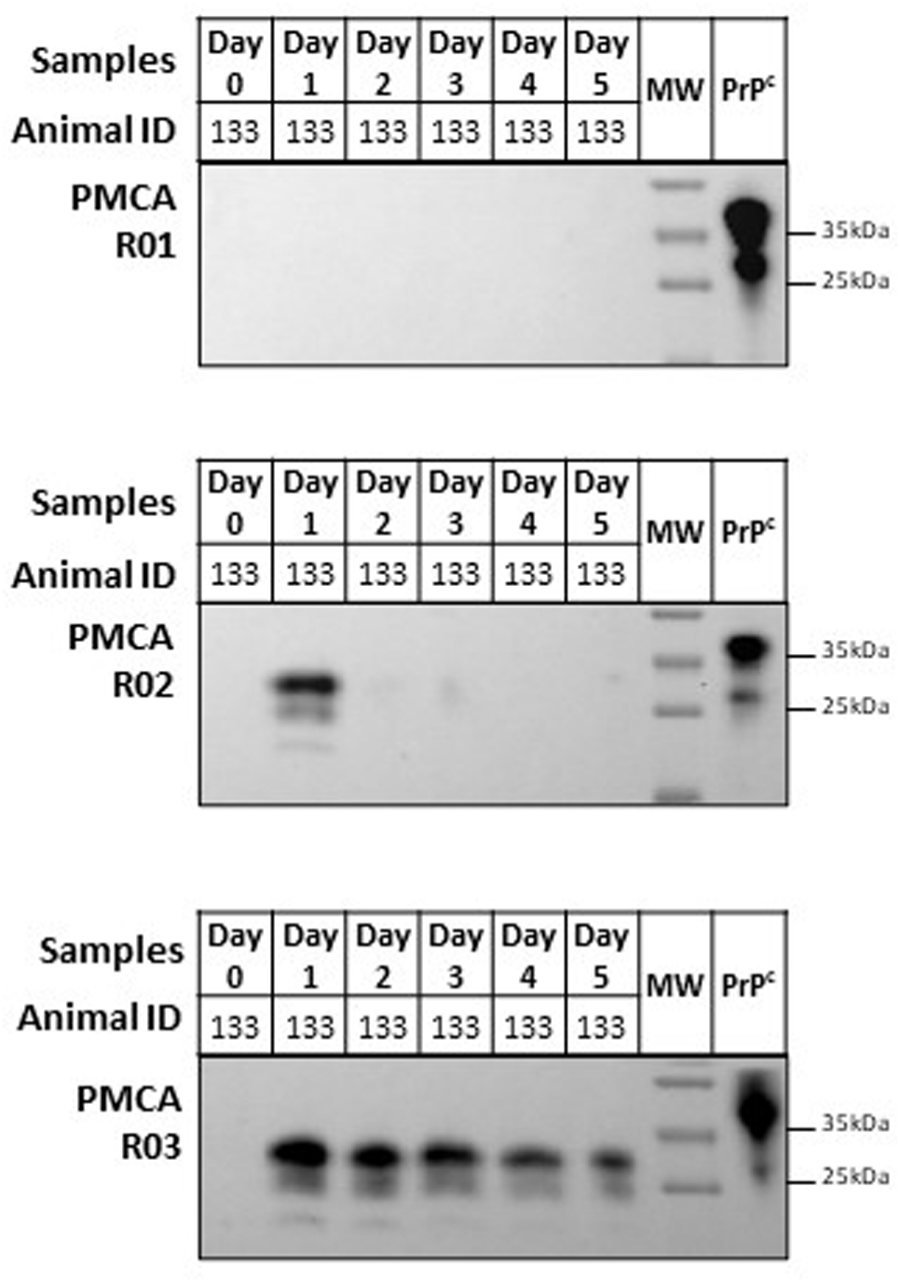
Representative data of serial PMCA for fecal samples from a coyote exposed to CWD prions. Representative analysis of fecal samples collected from Coyote #133 one day before (Day 0) and daily up to 5 days (Day 1-5) after exposing coyotes to CWD prions. All samples were treated with proteinase K, as explained in Material and Methods, with the exception of “PrP^C^” (brain extracts from tg1536 mice) that were used to control molecular weights and antibody reactivity. PMCA rounds are depicted at the left of each panel (R01-03). Numbers at the right represent molecular weights. The raw data for this figure is available in S2 Raw Image.

Our results confirmed the previous results communicated by Nichols *et al*. [23] which found no CWD prions in feces collected before exposure, or in feces collected from coyotes treated with the CWD-free brain (Coyote #132 and Coyote #134, Fig 2). In contrast, prion seeding activity was readily detected in feces from the coyotes fed with prion-infected brains (Coyote #133, Coyote #135 and Coyote #137, Fig 2). It is important to mention that CWD prions were only detected in feces after two or three PMCA rounds (Fig 3), irrespective of when the samples were collected. A single coyote (#133, Fig 3) provided signals at Day 1 in a second PMCA round, suggesting a higher load of prions at that specific time point. It is important to note that the previous study detected prions in coyotes’ feces only on days one and two after CWD exposure, demonstrating the increased sensitivity of the PMCA format used here. These changes in sensitivity may be due to different factors, including the addition of digitonin and Teflon beads that increase the sensitivity and reproducibility of this prion amplification method [28].

Previous reports by us [25] and others [24] used prion amplification assays to study the distribution of prions in animal tissues shortly after administration. Here, we tested nine separate tissue types (intestine, cecum, liver, tonsil, mesenteric lymph nodes, spleen, brain, lung and heart) from prion-exposed coyotes to explore similar paradigms. PMCA positive results were obtained from the cecum of the CWD-exposed animals, suggesting that prions are retained in this tissue several days after ingestion. The presence of CWD prions in the intestine of a single prion-exposed coyote (#135) further support the intestinal retention of ingested prions. Although gastrointestinal tissues were thoroughly washed to remove traces of ingested homogenate and feces from them, it is possible that some of the positive results observed were due to these materials. Nevertheless, positive signals were observed in tissues other than those of the gastrointestinal tract. Specifically, CWD-prions were also identified in the tonsils and mesenteric lymph nodes of single CWD-exposed coyotes (#137 and #135, respectively). These results suggest that CWD-prions were internalized after ingestion and reached some lymphoid tissues. This is consistent with a previous report showing that CWD prions readily migrate to lymph nodes in white-tailed deer [24]. Nevertheless, we acknowledge that the direct contact of prions with tonsils, due to ingestion, can explain the detection of CWD prions for this particular tissue. Another tissue showing positive prion detection was the lung (Fig 2). The detection in this tissue was obtained in only one of the three replicates tested for two different animals (#135 and #137). Importantly, all tissues from the coyotes fed with the CWD-free brains did not provide positive signals in our prion amplification assay (Fig 2).

## Discussion

Here, we confirmed previous findings [23] demonstrating that predators and scavengers can spread CWD prions via feces while retaining some infectious prions in their bodies. As previously suggested, these animals may act in a dual manner, by sequestering a low proportion of the infectious particles in their bodies while also disseminating transmission-relevant titers into the environment. We explored the fate of CWD prions after entering the coyotes’ bodies by testing multiple tissues five days after ingestion. We effectively found that a number of the tissues contained *in vitro* (PMCA) prion seeding activities, demonstrating that some of the ingested infectious particles are retained in these animals [21]. Although some studies show that prions can be degraded by elements of the digestive tract [31], others demonstrate that their binding to other particles such as soils protects them from this degradation [32]. Further studies should explore the potential role of gastrointestinal retention and degradation in the prion infectivity titers present in excreta and whether this varies in different animal species (specifically focused in ruminants and carnivore predators).

The role of wildlife species that share environments with cervids in the translocation of CWD via feces has been experimentally explored, as well as their ability to become infected by CWD prions. The latter creates a worrisome scenario as prions adapted in a new species may generate new prion strains of unknown infectivity and host ranges [7–9]. This possibility was not explored here, due to the short post-exposure periods of this study. Previous reports suggesting that canids are resistant to prion infection [33,34] makes this possibility unlikely. Regardless, our data demonstrate that a fraction of CWD prions ingested by coyotes are incorporated in their tissues, while larger levels are released in feces. The presumable reduction of prion titers after crossing the gastrointestinal tract of predators [23] suggest that the presence of coyotes in specific environments may be beneficial in reducing CWD prions. Along this line, the expanding geographic range of coyotes in North America [35] may result in additional benefits. However, the fact that infectious relevant levels of prions are released in coyotes’ feces several days post ingestion suggest that they might promote the environmental spreading of CWD. Considering this, evaluating the dual implications of the CWD-coyote interactions in the spread and prevalence of CWD will need substantial epidemiological and molecular research efforts.

It is important to clarify certain discrepancies between the findings found in the present study compared to those communicated in the original report [23]. First, the previous article by Nichols *et al*. used different PMCA settings compared to those used in the current study. Specifically, the current PMCA protocol results in a considerable higher sensitivity. This explains the fact that here we detected CWD-prions in feces for additional days after prion exposure compared to the first publication.

The results presented here provide additional information on the fate of CWD prions consumed by a canid mesopredator and sheds additional light on the potential role of cervid sympatric species, specifically predators, in the ecology of CWD.

## Supporting information

S1 Fig Additional replicates for samples represented in Fig 2. A) Serial amplification of a prion seed of known seeding activity (as described in Materials and Methods). This was used to test PMCA seeding efficiency. B) Unseeded PMCA reactions used as negative control. C-E) PMCA analysis of tissues from Coyote #137. Tissues like rectum, kidney, and some lymph nodes were uniquely collected for this animal and tested. However, as these tissues were not collected for all coyotes, they were not included in the formal analysis shown in Figure 2. PMCA positive tissues are depicted in red. All samples were treated with proteinase K, as explained in Material and Methods, with the exception of “PrP^C^” that was used to control molecular weights and antibody reactivity. Results shown in this figure correspond to a third PMCA round.
(PPTX)

S1 Raw Image Raw data for Fig 2. (JPG)

S2 Raw Image Raw data for Fig 3. (JPG)

S3 Raw Image Raw data for Supplementary Fig 1. (JPG)
