# Supplementary material for: Distribution of chronic wasting disease (CWD) prions in tissues from experimentally exposed coyotes (Canis latrans)
Source: PLoS One. 2025 Jul 9;20(7):e0327485. doi: 10.1371/journal.pone.0327485 (PMC12240315; doi:10.1371/journal.pone.0327485)
Supplement: S1 Fig — A) Serial amplification of a prion seed of known seeding activity (as described in Materials and Methods). This was used to test PMCA seeding efficiency. B) Unseeded PMCA reactions used as negative control. C-E) PMCA analysis of tissues from Coyote #137. Tissues like rectum, kidney, and some lymph nodes were uniquely collected for this animal and tested. However, as these tissues were not collected for all coyotes, they were not included in the formal analysis shown in Figure 2. PMCA positive tissues are depicted in red. All samples were treated with proteinase K, as explained in Material and Methods, with the exception of “PrPC” that was used to control molecular weights and antibody reactivity. Results shown in this figure correspond to a third PMCA round. (PPTX) [file pone.0327485.s001.pptx]

## Slide 1
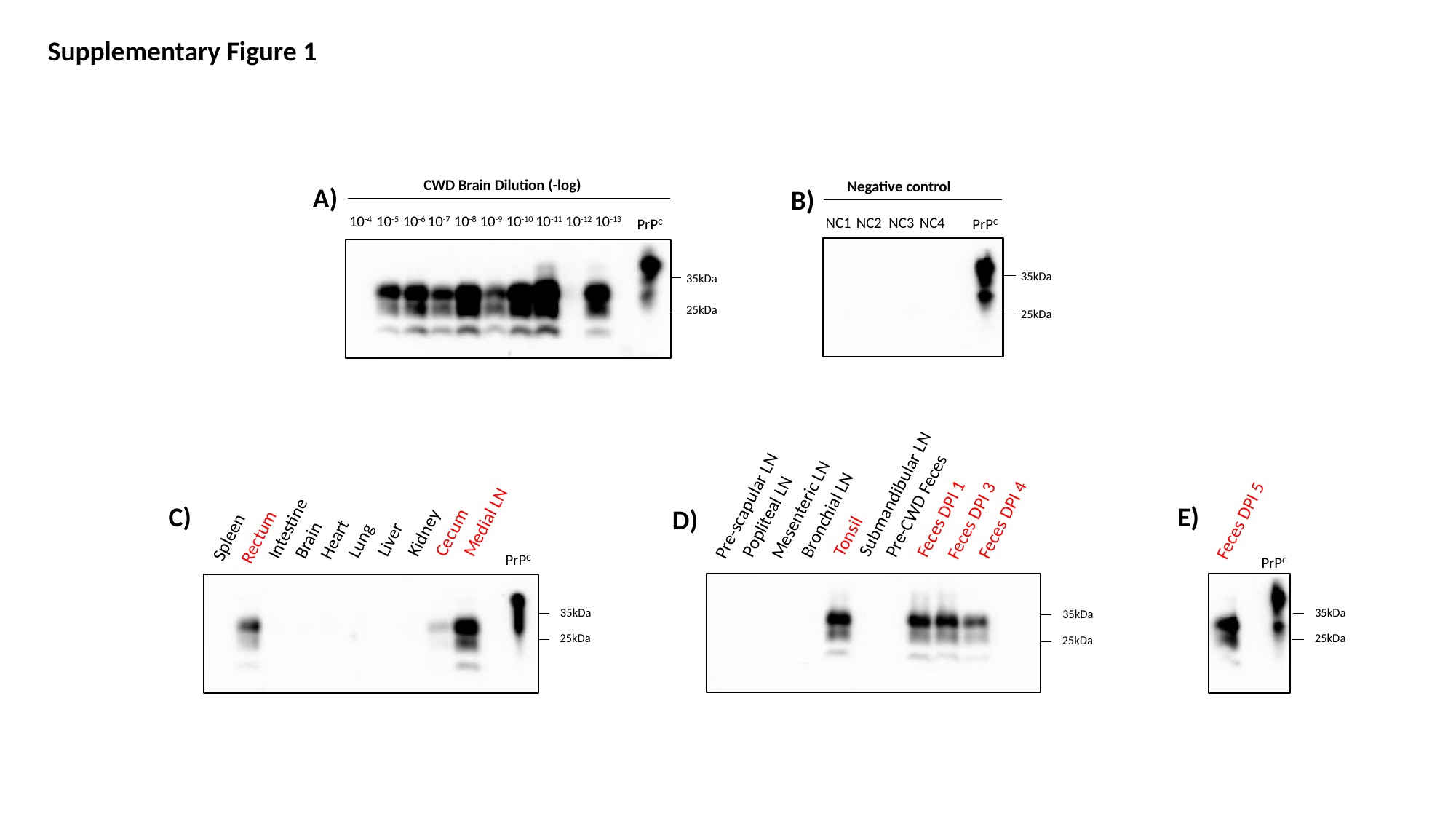

Supplementary Figure 1
CWD Brain Dilution (-log)
Negative control
A)
B)
10-11
10-4
10-5
10-6
10-7
10-12
10-10
10-9
10-8
10-13
NC4
NC1
NC3
NC2
PrPC
PrPC
35kDa
35kDa
25kDa
25kDa
Pre-CWD Feces
Tonsil
Intestine
Popliteal LN
Liver
Submandibular LN
Feces DPI 1
Feces DPI 5
Feces DPI 4
Bronchial LN
Pre-scapular LN
Mesenteric LN
Medial LN
C)
E)
D)
Feces DPI 3
Kidney
Cecum
Lung
Brain
Heart
Spleen
Rectum
PrPC
PrPC
35kDa
35kDa
35kDa
25kDa
25kDa
25kDa
