# Supplementary figures and images for: Distribution of chronic wasting disease (CWD) prions in tissues from experimentally exposed coyotes (Canis latrans)
Source: PLoS One. 2025 Jul 9;20(7):e0327485. doi: 10.1371/journal.pone.0327485 (PMC12240315; doi:10.1371/journal.pone.0327485)

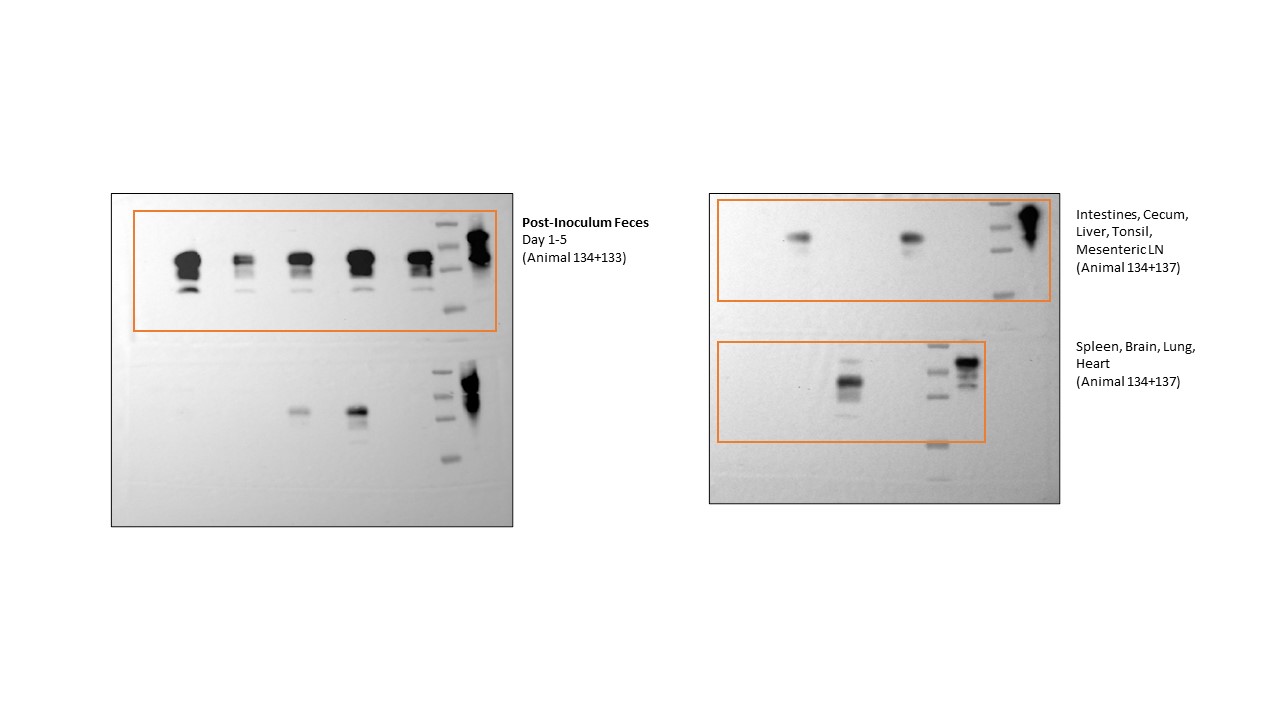

Supplement: S1 Raw Image — (JPG) [file pone.0327485.s002.jpg]

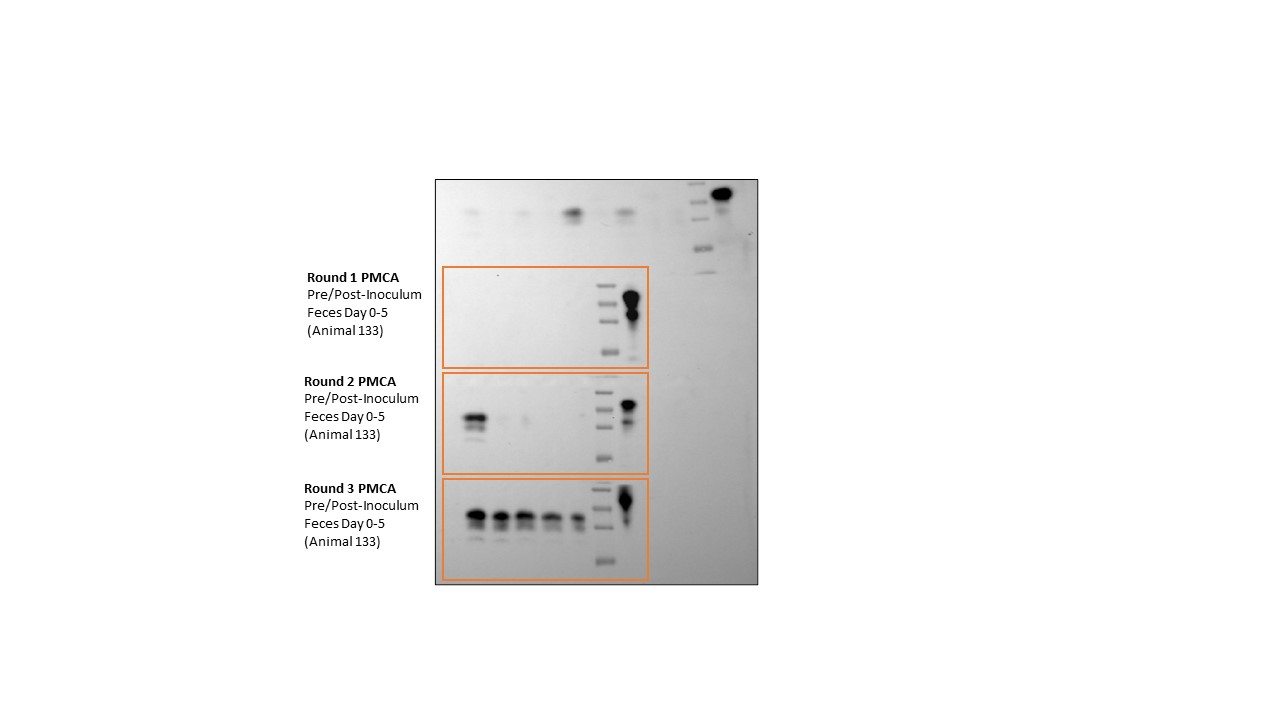

Supplement: S2 Raw Image — (JPG) [file pone.0327485.s003.jpg]

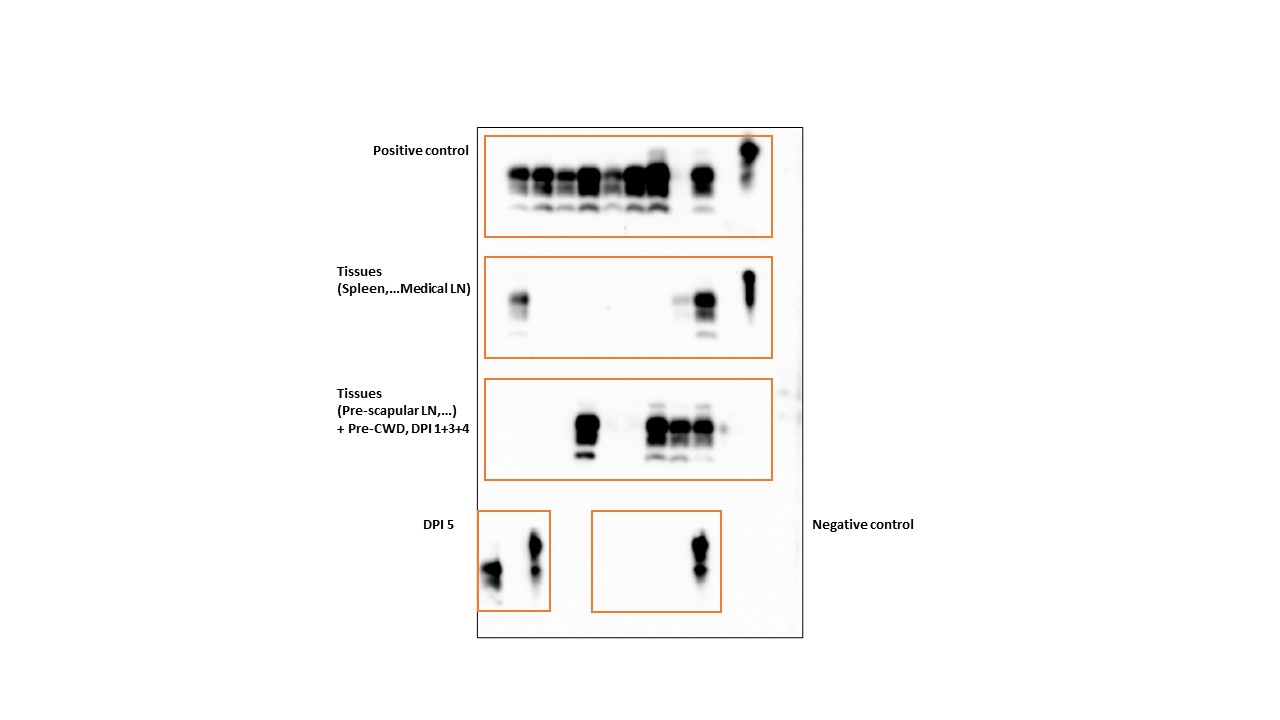

Supplement: S3 Raw Image — (JPG) [file pone.0327485.s004.jpg]
